# Effectiveness of Nurse-Led Interventions for the Prevention of Mental Health Issues in Patients Leaving Intensive Care: A Systematic Review

**DOI:** 10.3390/healthcare10091716

**Published:** 2022-09-07

**Authors:** Junpei Haruna, Takeshi Unoki, Nozomi Nagano, Shigeko Kamishima, Tomoki Kuribara

**Affiliations:** 1 Department of Intensive Care Medicine, School of Medicine, Sapporo Medical University, Sapporo 060-8543, Hokkaido, Japan; 2 Department of Acute and Critical Care Nursing, School of Nursing, Sapporo City University, Sapporo 060-0011, Hokkaido, Japan; 3 Department of Advanced Critical Care and Emergency Center, Sapporo Medical University Hospital, Sapporo 060-8543, Hokkaido, Japan; 4 Department of Nursing, Reiwa Health Sciences University, Fukuoka 811-0213, Fukuoka, Japan

**Keywords:** mental health disorders, nurse-led interventions, post-intensive care syndrome

## Abstract

This study aimed to evaluate the effectiveness of nurse-led interventions for the prevention of mental health disorders after intensive care unit discharge through a systematic review of the literature. The searches were conducted in the MEDLINE (via PubMed), CINAHL, PsycINFO, and Cochrane Library databases for studies pertaining to such interventions. Two independent reviewers analyzed the studies, extracted data, and assessed the quality of the evidence. Six eligible articles were identified, all of which were regarding post-traumatic stress disorder after intensive care unit discharge. Some of the interventions were conducted during the admission and some after the discharge. One study found that multimedia education during admission improved anxiety and depression one week after discharge. The remaining five studies concluded that nurse-led interventions did not prevent mental health disorders three months to one year after intensive care unit discharge. Our review revealed a paucity of research into the effectiveness of nurse-led interventions for the prevention of mental health disorders after intensive care unit discharge. The timing and the content of these interventions, and the adequate training of nurses, appear to be key factors. Therefore, multidisciplinary interventions are likely to be more effective than those led by nurses alone.

## 1. Introduction

Mental health disorders in patients after discharge from intensive care are an important issue. Specifically, post-traumatic stress disorder (PTSD), anxiety, and depression are common in patients after intensive care unit (ICU) discharge [1,2]. These morbidities are collectively referred to as post-intensive care syndrome (PICS) [3]. A previous systematic review has reported the rates of PTSD, anxiety, and depression in ICU survivors to be 19.8%, 43%, and 15.4%, respectively [4,5,6]. The risk factors for mental health disorders following ICU discharge include severe sepsis [7], acute respiratory distress, trauma, and hypoxemia [8,9,10]. Pre-existing mental illness, female sex, age < 50 years, a lower level of education, a history of alcohol abuse, sedative or analgesic use in the ICU, and upsetting experiences in the ICU [8,11,12,13,14,15,16] have also been found to increase the risk of psychiatric disorders. The clinical symptoms of PTSD that can be observed in patients after ICU discharge include flashbacks, hyperarousal, and severe anxiety [4]. These symptoms are also associated with a decreased quality of life (QOL) [1,17].

The common interventions that are used in order to prevent the development of mental health disorders after ICU discharge include adjusting sedation medications, managing delirium [18], optimizing sleep [19], and providing rehabilitation [20]. One approach that is used to reduce PICS is known as the ABCDEF bundle (assess, prevent, and manage pain; both spontaneous awakening and breathing trials; choice of analgesia and sedation; delirium assessment, prevention, and management; early mobility and exercise; family engagement/empowerment) [21,22]. Nurse-led interventions are another option, and these have received attention in several randomized controlled trials (RCTs), owing to their low cost and simplicity. However, a systematic review of the effects of nurse-led interventions towards preventing mental health disorders after ICU discharge has not previously been conducted. Nurses, especially those in the ICU, have a greater impact on the patients because they are directly involved in patient care and spend a substantial amount of time with these patients. The research question that we hypothesized was “do nurse-led interventions prevent mental illness in post-intensive care patients?”. The purpose of this study was to systematically review the research into the effects of nurse-led interventions for the prevention of mental health disorders after ICU discharge.

## 2. Materials and Methods

This systematic review was conducted in accordance with the Cochrane Handbook and was reported in the manner prescribed by the preferred reporting items for systematic reviews and meta-analyses (PRISMA) checklist [23]. The study protocol was registered in PROSPERO (CRD42021277827).

### 2.1. Research Question

The research question we evaluated was as follows: Do nurse-led interventions prevent mental illness in post-intensive care patients?

### 2.2. Inclusion and Exclusion Criteria

Nurse-led interventions were defined as those in which the intervention and patient interaction were provided by nurses only, regardless of any supervision by other professionals. The studies that were included in our review had the following criteria: (1) randomized controlled trials (RCTs), (2) included adult participants who were engaging in or had been recently discharged from ICU, (3) included only nurse-led interventions for the prevention of mental health illnesses, (4) not multidisciplinary approaches, and (5) peer-reviewed publications. Music therapy and ICU diaries were excluded because these interventions are performed not only by nurses but also physicians and other specialists. Eligible studies were not restricted by language to avoid language bias.

### 2.3. Search Strategies and Study Selection

We searched the following databases for eligible studies: MEDLINE (via PubMed), CINAHL, PsycINFO, and Cochrane Library. Additionally, we searched for ongoing trials registered on the World Health Organization international clinical trials registry platform on 22 September 2021. The key search terms used to identify potentially relevant studies are listed in Appendix A. We also attempted to identify additional relevant studies by manually searching the reference lists of the studies that were returned by the search and articles citing such studies (found on Google Scholar). If sufficient information was unavailable, we contacted the authors of the study. Two of the seven reviewers independently screened the titles and abstracts to identify potentially relevant studies. Two reviewers then independently assessed their eligibility based on a full-text review. Disagreements between the reviewers were resolved by discussion to reach a consensus.

### 2.4. Data Collection

We extracted data on the study design, year of publication, sample size, age range of participants, severity of illness, interventions, comparators, study-specific outcomes assessed, and the scales and cutoffs used. These included results of PTSD, anxiety, depression, and a mental component summary (MCS) of a 36-items short-form survey (SF-36). Outcome data were categorized according to the time after ICU discharge at which they were measured. The categories were 3–6 months, 7–12 months, and 1–2 years after ICU discharge. Data were recorded using an Excel spreadsheet, and data extraction was performed by two reviewers independently. To pool the results, data were extracted from individual studies in a dichotomous manner. The means and standard deviations were calculated for continuous variables and, in cases where data were unreported or unclear, the study authors were contacted.

For discrete variables, missing patient outcome data were entered according to a worst-case scenario, with all missing patient data in the two groups recorded to indicate that the intervention was ineffective, and that the patient was suffering from mental health issues. For continuous variables, missing values were left out. Therefore, we did not pool the studies that did not report continuous outcome variables for all of the eligible participants.

### 2.5. Study Risk-of-Bias Assessment

The revised Cochrane risk-of-bias tool for randomized trials (RoB 2, Version 15 March 2019) [24] was used to critically appraise the randomized controlled trials that were included in this study. This was completed by two reviewers; where discussions between them did not lead to a consensus, a third assessor was involved.

### 2.6. Measures of Effect

The primary outcome was PTSD within 3–6 months, 7 months to less than 1 year, and more than 1–2 years after ICU discharge. The secondary outcomes were anxiety, depression, and change in QOL 3–6 months, 7 months to less than 1 year, and more than 1–2 years after ICU discharge. Another secondary outcome was the MCS in the SF-36. The scale measuring each outcome was not limited, and the cutoffs were based on the definition of each study’s cutoff.

### 2.7. Synthesis Methods

We planned to conduct a meta-analysis only if the interventions were adequately homogeneous. As each study was performed using different intervention methods, no meta-analysis was performed.

### 2.8. Assessment of Reporting Bias

International clinical trial registry platforms were searched to identify any completed but unpublished trials [25].

## 3. Results

### 3.1. Study Selection

**Figure 1:**
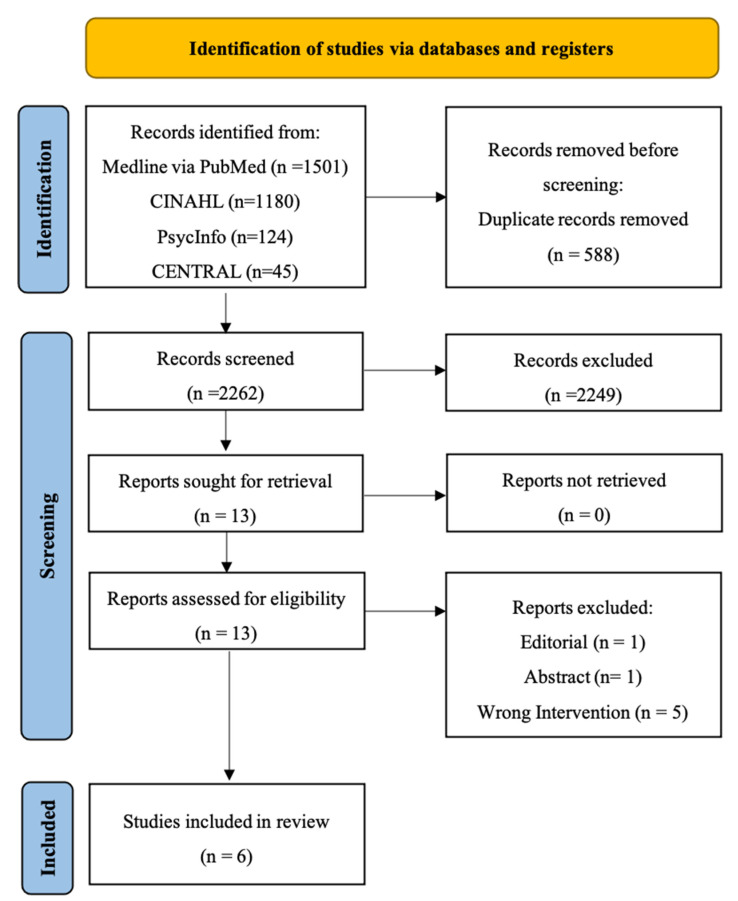
Preferred reporting items for systematic reviews and meta-analyses (PRISMA) diagram.

This systematic review was conducted according to the PRISMA checklist [23]. A PRISMA flowchart is shown in Figure 1. Our database search returned 2850 articles from PubMed (*n* = 1501), CINAHL (*n* = 1180), PsycINFO (*n* = 124), and the Cochrane Library (*n* = 45). The reference list was screened, but no additional papers were found. After the duplicate articles were removed (*n* = 588), the titles and the abstracts of the remaining articles were screened for their eligibility. This resulted in the exclusion of 2249 irrelevant papers. The full-text screening of the remaining thirteen papers further led to the exclusion of seven papers. Thus, six articles remained that met our criteria for final inclusion in our analysis, which was set for qualitative synthesis [26,27,28,29,30,31] (Figure 1).

### 3.2. Study Characteristics

In total, 1941 patients and six studies were qualitatively integrated (Table 1). The demographic and clinical characteristics of the study participants are shown in Table 1. 

The participants had spent time in ICUs for various reasons, including general surgery, cardiovascular surgery, coronary events, sepsis, and the need for mechanical ventilation. All six of the studies excluded patients with pre-existing cognitive impairments, mental health disorders, or delirium. One study excluded patients who had been hospitalized for head trauma, self-harm, or terminal illness. Some interventions took place while the patients were in the ICU [26,29,30] and others took place after ICU discharge [27,28,31]. The earliest post-ICU intervention was one week after discharge [31], and the latest intervention was one year after discharge [28]. The interventions could be classified into the following two types: preventative [26,28,29,31] and recovery-promoting [27,30] (Table 2).

The timing and the number of interventions varied, as did the methods that were used to measure the outcomes. The outcomes were evaluated between 3 and 12 months after ICU discharge. Each study used a different scale for outcome measurement, making it impossible to conduct a meta-analysis.

### 3.3. Study Design and Risk of Bias in the Included Studies

**Figure 2:**
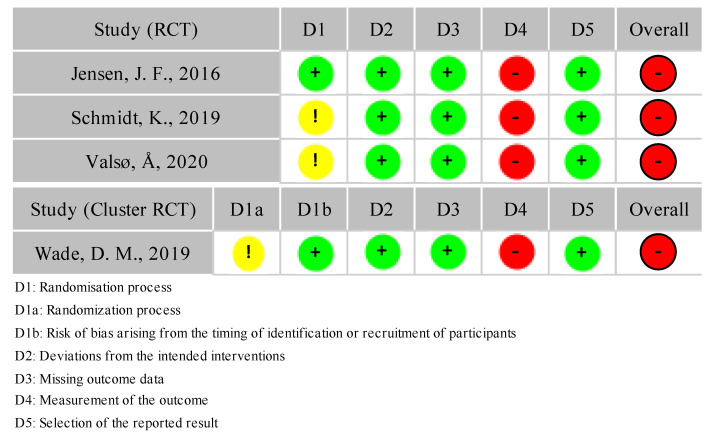
Risk of bias for PTSD.

The risk of bias was assessed for the predefined outcomes. Figure 2 shows each study’s risk of bias for the primary outcome of PTSD. The risks of bias for the secondary outcomes are shown in Appendix B. The ICTRP search resulted in 72 matches, however, no studies were unpublished.

For the primary outcome, three RCTs and one cluster RCT were included. Two of the three RCTs were blocked randomized trials. The risks of bias for the three RCTs are shown in Figure 2 [27,28,31]. All of the studies were rated as “high” for bias due to the outcome measurement because of the data that was obtained by self-reporting [27,31] or by telephone contact [28].

### 3.4. Primary and Secondary Outcomes

We were unable to conduct a meta-analysis due to the heterogeneity of the interventions; hence, we only performed a qualitative analysis. The effects of the interventions that were used in each study are described in Table 1. One study was a recovery program, one was multimedia education, and one was education in stress-management techniques. The manner in which these were delivered varied. The interventions that were provided in the other three studies were all nurse–patient consultations after ICU discharge. Three scales were used in order to assess PTSD, four to access anxiety, and two to access depression.

The only effective intervention was the multimedia education, which was conducted in the ICU [29]. This significantly reduced the anxiety and depression levels one week after ICU discharge. Aside from a multimedia education study, there were no considerable differences between the patients who received any interventions and the corresponding control group in the primary and secondary outcomes.

## 4. Discussion

This systematic review was conducted in order to evaluate the effectiveness of nurse-led interventions on the prevention of mental health disorders after ICU discharge. We identified six eligible articles that met our inclusion criteria. After reviewing these studies, we found that nurse-led interventions for PTSD after ICU discharge had no apparent effects, whether they were performed during the ICU admission or after the ICU discharge. We found that nurse-led interventions for anxiety and depression may reduce the incidence of these disorders in the short term, but they have no long-term effects. The lack of effect on the primary outcome may be attributable to the methodological differences between the studies.

One of the main methodological differences that was found in this review was the timing of the protocol initiation of the nurse-led interventions. It has been suggested that an early initiation of interventions for PTSD following time in an ICU may be helpful [32]. However, advanced interventions, such as cognitive behavioral therapy (CBT), when they are administered too early in an ICU stay may not be retained in the patient’s memory, owing to the patient’s physical problems [30]. Therefore, when nurse-led interventions are used in order to prevent PTSD, it is necessary to consider the timing of the intervention initiation carefully, taking into consideration the physical condition of the patient.

Most of the participants in the reviewed studies may have been at a low risk of mental health disorders after ICU discharge, which might have contributed to the results. The risk factors for PTSD after ICU discharge include delirium during the ICU admission [33,34], a high level of therapeutic intervention [35], and delusional memories [36,37]. This review found a lower severity of illness [38], prevalence of delirium [39], level of therapeutic intervention [40], and frequency of delusional memories [31] in the six studies compared to the studies of other interventions for mental health disorders after ICU discharge. Additionally, a recent meta-analysis suggested that older age was associated with the infrequency of PTSD development [16], although, the mean age of participants in all of the studies that were included in this review was older than 50 years. Moreover, two of the included studies had a lower prevalence of PTSD after ICU discharge compared to the previous studies [27,31]. The reason that the nursing intervention was not effective may have been the low baseline risk of mental illness after intensive care in these participants. Moreover, one of the assessed studies, which was conducted by Valsø et al. [31], identified a greater-than-average prevalence of patients with a high sense of coherence (SOC). Individuals with a high SOC have been shown to cope better with stress, and a high SOC is considered to be an important contributor to QOL [41]. It is possible that the participants in these studies were better able to prevent mental health disorders after ICU discharge using their own psychological resources. Therefore, nurse-led interventions might offer limited value to patients who are not at risk of mental health disorders after ICU discharge or those who are better able to cope with stress. The frequency of nurse-led interventions may also influence the results. The study in which the intervention consisted of follow-up consultations after ICU discharge provided 10+ interventions over about six months [42], whereas Valsø et al. [31] provided only 1–3 interventions over a short period after ICU discharge. In another study of interventions by clinical psychologists, which was conducted in order to improve PTSD and anxiety/depression after ICU discharge, the patients received five to six interventions during their ICU stay, which significantly reduced the prevalence of PTSD [43]. The most effective frequency of psychological interventions and follow-up after ICU discharge is still unknown; however, for most patients, follow-up should be ongoing and should be conducted at intervals that are individualized to each patient’s needs [44].

There are also several concerns about the quality of nurse-led interventions. In each of the studies that were assessed, the participants received several hours to several days of training prior to the nurse-led interventions. Those would be delivering the specialized therapies, such as CBT, must be well trained in both the professional and ethical guidelines and the specific techniques, with attention to the latest evidence in the field [45]. Therefore, the intervention quality must be above a certain level in order to be effective.

While nurse-led interventions can reduce the symptoms of anxiety and depression after ICU discharge in the short term, they do not improve the manifestation of the symptoms in the long term. The variation in the results between the studies likely depended on the timing of the anxiety and depression assessments. Although the nurse-led interventions were found to be ineffective against anxiety and depression three months after ICU discharge, some of the study participants had used other social resources for mental health support [27]. The previous reports suggest that discussing needs with a trusted healthcare professional after ICU discharge can help to prevent anxiety and depression [46]. Given that mental health issues correlate with physical recovery, it is important that rehabilitation is also used in conjunction [47]. Integrated interventions that include support from nurses and specialized ICU clinics may improve anxiety and depression [28]. We consider it important to utilize all available social resources in order to prevent long-term anxiety and depression.

No reliable measures for the prevention and treatment of mental health disorders after ICU discharge have been established at this time. A recent meta-analysis has found that ICU diaries do not reduce PTSD or anxiety [48]. Recently, there has been a focus on multidimensional interventions that incorporate peer and family support [49], multidisciplinary rehabilitation programs [50,51], PICS clinics [52], and medical management [2]. It is important to consider multidisciplinary interventions and multifaceted approaches rather than nurse-led approaches only.

### Strengths and Limitations

This study is the first systematic review of nurse-led interventions for the prevention of mental health disorders after ICU discharge. It has the advantage of including only RCTs that have been conducted in a qualified manner and includes studies in any language from any country. However, the study also has some limitations. First, it was not possible to conduct a meta-analysis because of the variety of the interventions in the included studies. Second, the definitions of the interventions were clinically heterogeneous.

## 5. Conclusions

This systematic review has examined the effectiveness of nurse-led interventions in preventing mental health disorders after ICU discharge. We have concluded that the current range of nurse-led interventions is ineffective for this purpose. However, stronger evidence for or against the effectiveness of these interventions is needed, and large multicenter studies should be conducted for this purpose, with standardized interventions of consistent duration, number, and frequency. The negative effects of mental health disorders after ICU discharge require the provision of appropriate support and treatment through a multi-professional approach.

## Figures and Tables

**Table 1:** Characteristics of the studies on the effectiveness of nurse-led interventions to prevent mental health impairment in post-intensive care patients.

Author, Year, Country	Target Population	Number of Patients I/C *n* (%)	Average Age I/C (years)	Male I/C *n* (%)	Outcome and Evaluation Scale
Fleischer, S., 2014, Germany [26]	Patients expected to stay more than 24 h in cardiac surgery, general surgery, and internal medicine	104 (49.3)/107 (50.7)	68/68	66 (63.5)/ 71 (66.4)	Anxiety: CINT score, STAI state, VAS-A
Jensen, J.F., 2016, Denmark, [27]	Adult patients with more than 48 h of MV	190 (49.2)/196 (50.8)	66/67.5	112 (58.9)/117 (59.7)	PTSD: HTQ-IV score, Anxiety: HADS, Depression: HADS, MCS: SF-36
Schmidt, K., 2016, Germany, [28]	Critically ill adult patients admitted to the ICU with sepsis	148 (50.9)/142 (49.1)	62.1/61.2	105 (70.9)/87 (61.3)	PTSD: PTSS-10 Depression: MDI MCS: SF-36
Demircelik, M.B., 2016, Turkey, [29]	Patients admitted to the coronary ICU who could communicate verbally and answer questionnaires	50 (50)/50 (50)	59/62	34 (68)/ 30 (60)	Anxiety: HADS Depression: HADS
Wade, D.M., 2019, the United Kingdom, [30]	Participants were critically ill patients who had received Level 3 (intensive) care and had regained mental capacity	314 (43.1)/415 (56.9)	60.4/57.2	187 (55.0)/268 (60.1)	PTSD: PSS-SR Anxiety: HADS Depression: HADS
Valsø, Å., 2020, Norway, [31]	Adult patients who stayed in the ICU for more than 24 h	111 (49.6)/113 (50.4)	53/50	50 (45)/ 55 (49)	PTSD: PTSS-10

I/C: Intervention/comparator; MV: Mechanical ventilator; HTQ-IV: Harvard Trauma Questionnaire Part IV; HADS: Hospital Anxiety and Depression Scale; CINT score: A Faces Scale for the assessment of anxiety in critically ill patients; STAI state: State and Trait Anxiety Inventory; VAS-A: Visual analog scale-anxiety; PTSS-10: Post-Traumatic Stress Scale 10; MDI: Major Depression Inventory; PSS-SR: PTSD Symptom Scale–Self-Report; MCS: Mental component summary; SF-36: MOS 36-item short-form health survey; ICU: Intensive care unit.

**Table 2:** Nurse-led interventions and outcomes from studies.

Author, Year	Intervention	Evaluation Scale	Measurement Timing ^†^1	Findings, Results
Fleischer, S, 2014 [26]	Lazarus’ cognitive mediational theory of stress and emotion was applied and implemented during the ICU stay.	Anxiety: CINT score	Unknown	The results of the CINT score showed that the intervention group had a mean of 20.4 (SD 14.4) and the control group had a mean of 20.8 (SD 14.7). The results of the VAS-A were a mean of 12.7 (SD 18.6) for the intervention group and a mean of 11.9 (SD 17.4) for the control group, indicating a significant decrease in anxiety. However, the results measured by all of the scales showed no significant decrease in anxiety.
		Anxiety: STAI state	Unknown	
		Anxiety: VAS-A	Unknown	
Jensen, J.F, 2016 [27]	Three consultations and recovery programs after ICU discharge.	PTSD: HTQ-Ⅳ score	3~6 months, 1~2 years	HTQ-IV scores for 3–6 months after ICU discharge showed 35 events (incidence 38.8%) in the intervention group and 45 events (incidence 32.5%) in the control group. The results for 1–2 years after ICU discharge showed 35 events (incidence 30.1%) in the intervention group and 35 events (incidence 32.1%) in the control group. There was no intervention effect at 3–6 months or 1–2 years after ICU discharge.
		Anxiety: HADS	3~6 months, 1~2 years	HADS scores for 3–6 months after ICU discharge showed 12 events (incidence 8.8%) in the intervention group and 22 events (incidence 16.1%) in the control group, and those for 1–2 years after ICU discharge showed 12 events (incidence 9.1%) in the intervention group and 13 events (incidence 10%) in the control group. There was no intervention effect at 3–6 months or 1–2 years after ICU discharge.
		Depression: HADS	3~6 months, 1~2 years	HADS scores for 3–6 months after ICU discharge showed 11 events (incidence 8.0%) in the intervention group and 16 events (incidence 11.7%) in the control group, and those for 1–2 years after ICU discharge showed 12 events (incidence 9.2%) in the intervention group and 11 events (incidence 8.4%) in the control group. There was no intervention effect at 3–6 months or 1–2 years after ICU discharge.
		MCS: SF-36	3~6 months, 1~2 years	SF-36 MCS scores for 3–6 months after ICU discharge showed a mean of 51.9 (SD 16.2) in the intervention group and a mean of 49.94 (SD 16.4) in the control group, and those for 1–2 years after ICU discharge showed a mean of 48.8 (SD 12.5) in the intervention group and a mean of 49.2 (SD 12.6) in the control group. There was no intervention effect at 3–6 months or 1–2 years after ICU discharge.
Schmidt, K, 2016 [28]	The case management that nurses were trained to provide after ICU discharge.	PTSD: PTSS-10	3~6 months	PTSS-10 scores for 3–6 months after ICU discharge showed a mean of 24 (SD 23.1) in the intervention group and a mean of 23.2 (SD 9.7) in the control group.
		Depression: MDI	3~6 months	MDI scores for 3–6 months after ICU discharge showed 36 events (incidence 24.3%) in the intervention group and 32 events (incidence 22.5%) in the control group.
		MCS: SF-36	3~6 months	SF-36 MCS scores for 3–6 months after ICU discharge showed a mean of 48.8 (SD 12.5) in the intervention group and a mean of 49.2 (SD 12.6) in the control group.
Demircelik, M.B, 2016 [29]	Multimedia nursing education provided during ICU admission.	Anxiety: HADS	During ICU stay of one week	HADS scores during the ICU stay showed a mean of 6.1 (SD 0.7), CG showed a mean of 5.7 (SD 0.6), and for one week after ICU discharge the mean was 1.9 (SD 0.2) in the intervention group, and 5.1 (SD 0.6) in the control group.
		Depression: HADS	During ICU stay of one week	HADS scores during the ICU stay showed a mean of 5.4 (SD 0.6), CG showed a mean of 5.1 (SD 0.5), and for one week after ICU discharge the mean was 1.9 (SD 0.25) in the intervention group, and 4.8 (SD 0.5) in the control group.
Wade, D.M, 2019 [30]	Three stress support sessions and a relaxation and recovery program, which were conducted by trained ICU nurses during the ICU stay.	PTSD: PSS-SR	3~6 months	PSS-SR scores for 3–6 months after ICU discharge showed 75 events (incidence 23.8%) in the intervention group and 73 events (incidence 17.5%) in the control group. There was no intervention effect at 3–6 months after ICU discharge.
		Anxiety: HADS	3~6 months	HADS scores for 3–6 months after ICU discharge showed 121 events (incidence 38.5%) in the intervention group and 140 events (incidence 33.7%) in the control group. There was no intervention effect at 3–6 months after ICU discharge.
		Depression: HADS	3~6 months	HADS scores for 3–6 months after ICU discharge showed 104 events (incidence 33.1%) in the intervention group and 130 events (incidence 31.3%) in the control group. There was no intervention effect at 3–6 months after ICU discharge.
Valsø, Å, 2020 [31]	Nurse-led follow-up consultations after discharge from the ICU.	PTSD: PTSS-10	3~6 months	PTSS-10 scores for 3–6 months after ICU discharge showed a mean of 32 (SD 16.3) in the intervention group and a mean of 32 (SD 14.36) in the control group. There was no intervention effect at 3–6 months after ICU discharge.
			7 months to less than one year	PTSS-10 scores for 7 months to less than 1 year after ICU discharge showed a mean of 31 (SD 13.9) in the intervention group and a mean of 30 (SD 14.36) in the control group. There was no intervention effect at 3–6 months after ICU discharge.
			More than 1– 2 years	PTSS-10 scores for 3–6 months after ICU discharge showed a mean of 32 (SD 16.3) in the intervention group and a mean of 29 (SD 16.8) in the control group. There was no intervention effect at 3–6 months after ICU discharge.

HTQ-Ⅳ: Harvard Trauma Questionnaire Part IV; HADS: Hospital Anxiety and Depression Scale; CINT score: A Faces Scale for the assessment of anxiety in critically ill patients; STAI state: State and Trait Anxiety Inventory; VAS-A: Visual analog scale-anxiety; PTSS-10: Post-Traumatic Stress Scale 10; MDI: Major Depression Inventory; PSS-SR: PTSD Symptom Scale–Self-Report; ICU: Intensive care unit; MCS: Mental component summary; SF-36: MOS 36-item short-form health survey ^†^1. The period after ICU discharge is indicated.

## Data Availability

Not applicable.

## Appendix A. Search Strategy

CINAHL

#1	MH “intensive care unit”
#2	MH “critical care”
#3	MH “critical illness”
#4	TI “intensive care unit”
#5	AB “intensive care unit”
#6	TI “critical care”
#7	AB “critical care”
#8	TI “critical illness”
#9	AB “critical illness”
#10	TI “critically ill*”
#11	AB “critically ill*”
#12	TI ICU
#13	AB ICU
#14	MH nurse
#15	TI nurs*
#16	AB nurs*
#17	MH “Randomized Controlled Trials”
#18	PT “randomized controlled trial”
#19	TI “Randomized Controlled Trials”
#20	AB “Randomized Controlled Trials”
#21	MH “Clinical Trials”
#22	TI “clinical trial”
#23	AB “clinical trial”
#24	TI randomized
#25	AB randomized
#26	TI placebo
#27	AB placebo
#28	TI “clinical controlled trial”
#29	AB “clinical controlled trial”
#30	TI randomly
#31	AB randomly
#32	TI trial
#34	(MH “Animals”)
#35	(MH “Human”)
(#1 OR #2 OR #3 OR #4 OR #5 OR #6 OR #7 OR #8 OR #9 OR #10 OR #11 OR #12 OR #13) AND (#14 OR #15 OR #16) AND ((#17 OR #18 OR #19 OR #20 OR #21 OR #22 OR #23 OR #24 OR #25 OR #26 OR #27 #28 OR #29 OR #30 OR #31 OR #32 OR #33) NOT (#34 NOT #35)).

Cochran library

#1	MeSH descriptor: [Intensive Care Units] explode all trees
#2	(intensive care units):ti,ab,kw
#3	MeSH descriptor: [Critical Illness] this term only
#4	(critical illness):ti,ab,kw
#5	(“critically ill”):ti,ab,kw
#6	(“critically ill patients”):ti,ab,kw
#7	MeSH descriptor: [Critical Care] this term only
#8	(“critical care”):ti,ab,kw
#9	(ICU):ti,ab,kw
#10	MeSH descriptor: [Nursing] this term only
#11	MeSH descriptor: [Nurses] this term only
#12	(nurse):ti,ab,kw
#13	(nurses):ti,ab,kw
#14	(nursing):ti,ab,kw
#15	MeSH descriptor: [Randomized Controlled Trial] explode all trees
#16	(randomized controlled trial):pt
#17	(controlled clinical trial):pt
#18	(placebo):ti,ab,kw
#19	MeSH descriptor: [Clinical Trials as Topic] this term only
#20	(randomly):ti,ab,kw
#21	(trial):ti,ab,kw
#22	MeSH descriptor: [Animals] this term only
#23	MeSH descriptor: [Humans] this term only
(#1 OR #2 OR #3 OR #4 OR #5 OR #6 OR #7 OR #8 OR #9 OR #10 OR #11 OR #12 OR #13) AND (#14 OR #15 OR #16) AND ((#17 OR #18 OR #19 OR #20 OR #21 OR #22 OR #23 OR #24 OR #25 OR #26 OR #27 #28 OR #29 OR #30 OR #31 OR #32 OR #33) NOT (#34 NOT #35)).

PsycInfo

#1	TI”intensive care units”
#2	AB”intensive care units”
#3	TI”intensive care unit”
#4	AB”intensive care unit”
#5	TI”critical illness”
#6	AB”critical illness”
#7	TI”critically ill”
#8	AB”critically ill”
#9	TI“crically ill patients”
#10	AB”critically ill patients”
#11	TI “ICU”
#12	AB”ICU”
#13	DE nurses
#14	TI nurs*
#15	AB nurs*
#16	DE ”randomized controlled trials” OR DE ”randomized clinical trials” OR DE “clinical trials” OR DE “placebo”
#17	PT “randomized controlled trials”
#18	PT “controlled clinical trials”
#19	TI randomized
#20	AB randomized
#21	TI placebo
#22	AB placebo
#23	DE “clinical trial as topic”
#24	TI randomly
#25	AB randomly
#26	TI trial
#27	DE animals
#28	DE humans
(#1 OR #2 OR #3 OR #4 OR #5 OR #6 OR #7 OR #8 OR #9 OR #10 OR #11 OR #12) AND (#13 OR #14 OR #15) AND ((#16 OR #17 OR #18 OR #19 OR #20 OR #21 OR #22 OR #23 OR #24 OR #25 OR #26) NOT (#27 NOT #28)).

MEDLINE via PubMed

#1	intensive care units[MeSH]
#2	intensive care unit[Title/Abstract]
#3	Criticallyill* [Title/Abstract]
#4	critical illness[MeSH Terms]
#5	critical illness[Title/Abstract]
#6	Critical care[Title/Abstract]
#7	Critical care [MeSH Terms: noexp]
#8	ICU[Title/Abstract]
#9	nurse[MeSH Terms: noexp]
#10	Nurs*[Title/Abstract]
#11	randomized controlled trial”[Publication Type]
#12	controlled clinical trial”[Publication Type]
#13	“randomized”[Title/Abstract]
#14	placebo[Title/Abstract]
#15	clinical trials as topic[MeSH Terms: noexp]
#16	“randomly”[Title/Abstract]
#17	“trial”[Title]
#18	“animals”[MeSH Terms]
#19	“humans”[MeSH Terms]
(#1 OR #2 OR #3 OR #4 OR #5 OR #6 OR #7 OR #8) AND (#9 OR #10) AND ((#11 OR #12 OR #13 OR #14 OR #15 OR #16 OR #17) NOT (#18 NOT #19)).
